# Wine without water: Improving grapevine tolerance to drought

**DOI:** 10.1093/plphys/kiac381

**Published:** 2022-08-18

**Authors:** Alexandra J Burgess

**Affiliations:** Division of Agriculture and Environmental Sciences, School of Biosciences, University of Nottingham, Nottingham, LE12 5RD, UK

Improving crop productivity is a key aim of agricultural production. With a changing environment, this must be achieved through sustainable approaches, minimizing the effect of climate change on crop growth and productivity and maximizing the ability for a plant to acclimate to, or tolerate, the change in conditions (Rosenzweig and Hillel, 2008). The Intergovernmental Panel for Climate Change (IPCC) has indicated that substantial climate change has already occurred since the 1950s (IPCC, 2022). Global mean surface temperatures are projected to rise by upto 3.1°C by the end of the century. This temperature change, and the associated changes to precipitation, are expected to increase the frequency and severity of droughts, in turn influencing crop yields (Hatfield et al., 2011).

Maintaining productivity under drought is usually achieved through careful selection of genotype combined with management practices that maximize water availability and minimize water use. In terms of species or varietal selection, drought tolerance is determined by an integrated set of traits including, but not limited to, stomatal conductance and closure dynamics, accumulation of solutes, turgor pressure, and xylem vulnerability to embolism (i.e. air bubbles) (Figure 1; Blackman et al., 2019). Whilst variation in each of the traits can be seen, the optimal combinations of traits facilitating drought tolerance under present and future climate scenarios are not known.

**Figure 1:**
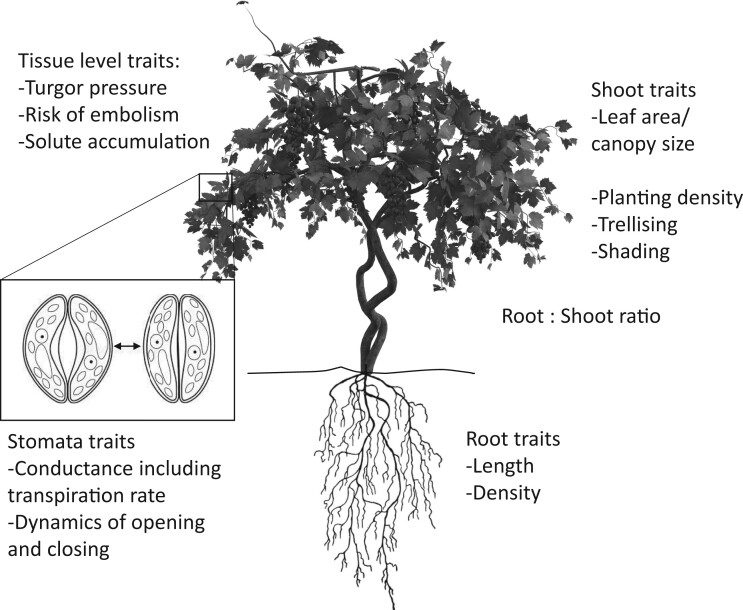
Overview of traits contributing to drought tolerance in grapevine and other land plants.

Within this issue, Dayer et al. (2022) present a modeling approach to predict the optimal trait combinations conveying drought tolerance in grapevine (*Vitis sp.*). By quantifying eight hydraulic traits that contribute to drought tolerance across nine diverse *Vitis* species and cultivars, the authors created in silico libraries of trait combinations by rearranging the quantified traits at random. The resultant combinations were assessed using the hydraulic model “Sureau” by simulating the time taken for a mature vineyard to reach 100% loss of conductivity under drought (Dayer et al., 2020). From this, the authors identified the 200 best performing combinations using currently available variability, which they termed the “Elites,” and the 200 best performing combinations using the current variability ±50%, termed the “Super Elites.” Many of the Elites and Super Elites exhibited a drought-tolerant trait syndrome with reduced transpiration and increased hydraulic safety margins (HSM). However, reduced transpiration and increased HSM were not ubiquitous to all syndromes, indicating a diversity in trait combinations with no single trait, or traits, conveying superior performance under drought. Other drought tolerance traits included those that reduce canopy surface area and increase rooting area, which can partly be achieved through management practices such as reduced planting density or trellising.

In addition to identification of potentially high-performing trait syndromes, Dayer et al. (2022) simulated crop performance across six global wine regions. Under wetter and cooler regions of France, existing *Vitis* genotypes were predicted to maintain productivity until 2070, whilst in Spain existing genotypes were predicted to suffer loss after 2050, with Elite varieties suffering after 2070. However, in California, no current genotypes were suitable for growth without additional inputs of water, and even Super Elite varieties were expected to suffer losses after 2080 in the Paso Robles region. Thus, the presented modeling approach provides two purposes: to identify potentially productive trait combinations and to indicate growing regions where additional inputs, such as irrigation, may be required.

For perennial crops such as grapevine, substantial amounts of time and money are required for the development of new varieties. Furthermore, due to the high rate of vegetative propagation from a restricted set of elite cultivars, the available genetic diversity within commercially grown perennial crops is limited (Miller and Gross, 2011). Even for annual crops, a targeted approach to cultivar development, such as that presented by Dayer et al. (2022), combined with speed-breeding methodologies would substantially contribute to stabilizing and improving productivity under future scenarios (Hickey et al., 2019). This is particularly important for multi-trait systems such as drought tolerance, whereby utilizing existing variation and the emergent properties of novel trait combinations could provide a partial solution to preparing agricultural systems for climate change.

## Funding

AJB is supported by The Leverhulme Trust as an Early Career Fellow.

*Conflict of interest statement.* None declared.
